# 

**Published:** 1913-05

**Authors:** 


### CONTENTS.

ORIGINAL COMUNICATIONS—

The Treatment of Chorea with Rheumatism Phylacogen.
By E. Bates Block, M. D., Atlanta, Ga. ............ 49

Mortality and Prevalence of Typhoid Fever. By
Lewis M. Gaines, B. S., M. D., Atlanta, Ga. ...... 54

EDITORIAL ........................................ 61

SELECTIONS AND ABSTRACTS .................. 64

